# The Course of Neurocognitive Changes in Acute Psychosis: Relation to Symptomatic Improvement

**DOI:** 10.1371/journal.pone.0167390

**Published:** 2016-12-15

**Authors:** Liss Anda, Kolbjørn S. Brønnick, Erik Johnsen, Rune A. Kroken, Hugo Jørgensen, Else-Marie Løberg

**Affiliations:** 1 TIPS, Division of Psychiatry, Stavanger University Hospital, Stavanger, Norway; 2 Department of Biological and Medical Psychology, University of Bergen, Bergen, Norway; 3 Institute of Health, Faculty of Social Sciences, University of Stavanger, Stavanger, Norway; 4 Division of Psychiatry, Haukeland University Hospital, Bergen, Norway; 5 Department of Clinical Medicine, University of Bergen, Bergen, Norway; 6 Department of Clinical Psychology, University of Bergen, Bergen, Norway; 7 Department of Addiction Medicine, Haukeland University Hospital, Bergen, Norway; SPAIN

## Abstract

**Introduction:**

Cognitive impairment is a core aspect of psychosis, but the course of cognitive functioning during acute psychosis remains poorly understood, as does the association between symptom change and neurocognitive change. Some studies have found cognitive improvement to be related to improvement in negative symptoms, but few have examined cognitive changes in the early acute phase, when clinical improvement mainly happens. This study’s aim was to investigate the relation between cognitive and symptomatic change in clinically heterogeneous patients during the early acute phase of psychosis.

**Method:**

Participants (n = 84), including both first-episode and previously ill patients, were recruited from consecutive admissions to the acute psychiatric emergency ward of Haukeland University Hospital, Bergen, Norway, as part of the Bergen Psychosis Project (BPP). The RBANS neurocognitive test battery was administered on admission and again at discharge from the acute ward (mean time 4.1 weeks, SD 1.86 weeks). Symptomatic change was measured by PANSS.

**Results:**

The proportion of subjects with cognitive impairment (t < 35) was 28.6% in the acute phase and 13.1% at follow-up. A sequential multiple linear regression model with RBANS change as the dependent variable found PANSS negative symptoms change to significantly predict total RBANS performance improvement (beta = -.307, p = .016). There was no significant difference between subjects with schizophrenia and those with other psychotic disorders in terms of cognitive change.

**Conclusion:**

The proportion of subjects with mild to moderate impairment in cognitive test performance is reduced across the acute phase of psychosis, with improvement related to amelioration of negative symptoms.

## Introduction

Cognitive impairment is a core characteristic of schizophrenia and related psychoses [1, 2], and cognitive functioning is important to successful recovery from psychosis [3, 4]. Cognitive impairment arises ahead of first episode psychosis, [5] is evident even at primary school age [6] and further deteriorates into adulthood [7]. Negative and disorganized symptoms have been associated with neurocognitive impairment [8–12]. However, the trajectory of cognitive functioning after onset of psychosis remains unclear [3], as does the relation between cognition and clinical symptoms. Some have found cognitive functioning to remain largely unchanged after the onset of psychosis [13–17], while others argue that cognitive performance further declines over time in chronically ill patients, with processing speed, learning and executive functioning showing particular impairment [3, 7]. In contrast to findings of continued impairment, a recent comprehensive meta-study also found cognition to improve modestly with treatment, both in groups deemed at ultra high risk of developing psychotic disorders, and in first episode psychosis groups [18], an effect also found in non-first episode samples [19]. It is also possible that different functional dimensions of cognition have different trajectories, with some static and others dynamic over time [20]

Given that the acute psychotic phase sees the greatest symptom alleviation and early treatment effects, the most pronounced cognitive changes might also take place alongside these other processes. However, most studies on cognition in psychosis have focused on stabilized phases of psychotic illness, which is sub-optimal with regard to assessing the relation between symptomatic change and cognitive dysfunction. As a result, the relation between symptomatic and cognitive change remains underexplored. One study, looking specifically at changes in executive functioning during the first four weeks of acute hospital admission for schizophrenia, indeed found an improvement in cognitive performance, but unrelated to symptom load change [21]. A later study, also of acutely admitted subjects, found working memory to improve over a period of four weeks, related to negative symptom load [22]. However, neither of these studies directly compared cognitive change with changes in psychotic symptom severity. A third study with retesting done at six weeks found that amelioration of negative symptoms predicted improvement in working and verbal learning scores, while overall symptomatic improvement predicted better performance in verbal learning [23]. Notably, one recent study where first-episode psychosis patients were re-tested after 12 weeks, found an improvement in overall cognition, working memory and verbal learning, with cognitive improvement mediated by symptom improvement [24]. However, most research looking at cognitive variables in psychosis has performed cognitive assessment at various times later in the acute phase, collecting baseline data up to two weeks after admission[25, 26], or even as late as 3 months [27, 28] or 6 months [6]. This means that the course and nature of cognitive impairment and its relationship to symptomatic change during the acute phase of psychosis remains particularly poorly understood.

In addition to focusing on stable phases of illness, studies have mainly investigated homogenous patient groups with schizophrenia (e.g. [13, 20, 29–31]). It has been suggested that subjects with schizophrenia show more severe neurocognitive impairment and a less favourable trajectory for cognitive change than do subjects with other psychotic disorders [10], meaning that restrictive sampling might limit variability and ecological validity. The present study therefore aims to investigate the course of naturally occurring changes in cognitive performance seen during the early acute phase of psychosis, in a heterogeneous sample of subjects consecutively admitted to an acute mental health ward. We also wished to examine any correlation between cognitive change and changes in symptom load during this stage of illness, as well as effects of diagnostic group.

We hypothesize that 1) overall cognitive performance will be impaired during the acute phase of psychosis, 2) there will be improvement in cognitive performance during the early acute phase of psychosis, which will be related to an improvement in negative symptoms across this time period, and that 3) the cognitive performance of participants with a diagnosis of schizophrenia will show less improvement than that of participants with other psychotic disorders.

## Methods

### Study design

Participants were recruited from consecutive admissions to the acute psychiatric emergency ward of Haukeland University Hospital, Bergen, Norway, as part of the Bergen Psychosis Project (BPP), a 24-month prospective, rater-blinded, pragmatic, randomized, comparison of first-line second-generation antipsychotics. The project was approved by the Norwegian Social Science Data Services and the Regional Committee for Medical Research Ethics (RCMRE), whom allowed eligible patients to be included before providing informed consent, aiming to achieve a clinically relevant representation of patients with acute psychosis. At first follow-up, all participants were asked for written informed consent to participate in the follow-up project. The BPP was independently funded, and is described in greater detail in Johnsen et al. [32].

### Subjects

Inclusion criteria for the BPP were age ≥ 18, a score of ≥ 4 on one or more of the items Delusions, Hallucinatory behaviour, Grandiosity, Suspiciousness/persecution, or Unusual thought content in the Positive and Negative Syndrome Scale (PANSS) [33], ability to understand Norwegian language, and eligibility for oral antipsychotic drug therapy. Patients met ICD-10 [34] criteria for schizophrenia, schizoaffective disorder, schizophreniform disorder, brief psychotic episode, delusional disorder, drug-induced psychosis, and major depressive disorder with psychotic features, as determined by experienced clinicians. Exclusion criteria were ineligibility for oral antipsychotics, organic brain disease, manic psychosis, substance-induced psychosis resolving within a few days of admission, inability to cooperate reliably during investigations, indication for electroconvulsive therapy, or being medicated with clozapine on admittance. Participants involved in the current project were included between March 2004 and January 2009.

Participants from the main BPP study were included in the current sub-study if they had completed both a PANSS interview and neurocognitive testing both at baseline and at first follow-up, yielding a total N = 84, aged on average 33.3 years and including 32.1% females. Sociodemographic variables are displayed in Table 1.

**Table 1:** Sociodemographic data for the sample (N = 84).

*Mean age (SD)*	*33.3 (13.3)*
Years education (SD)	12.5 (2.6)
Baseline GAF functioning (SD)	30.5 (4.8)
Females %	32.1
Ethnicity white %	90.5
First hospital admission %	56.5
Previously never used antipsychotics %	51.2

All values reported as mean (standard deviation), except where noted.

### Measures

Baseline assessments were performed immediately after admittance to the acute psychiatric ward. The first follow-up, which formed the basis for comparisons used in the present study, was performed at the time of acute ward discharge or after 6 weeks, which ever came first.

### Cognitive assessments

Cognitive performance was assessed using the Repeatable Battery for the Assessment of Neurocognitive Status (RBANS) [35]. RBANS is sensitive to typical cognitive deficits in schizophrenia, and has shown good validity and reliability [36–38]. It measures global performance as well as the sub-domains of Visuospatial/Constructional Ability, Language, Attention, and Immediate as well as Delayed Memory. Administering the RBANS takes only approximately 30 minutes, which makes it feasible to use when testing acutely ill subjects. RBANS exists in two separate forms with norm data adjusted for re-test effects [37], making it suitable for repeated testing with the aim of detecting cognitive change. Follow-up testing was done using the alternate form of RBANS. The overall RBANS score is considered to have good test–retest reliability (Gold et al., 1999 and Wilk et al., 2002). Scores on the Norwegian research version of the RBANS have been shown to be valid when compared to results from a comprehensive test battery [39]. Tests were administered by trained research nurses.

### Clinical assessments

Symptoms were assessed at baseline and follow-up using the PANSS [33]. For the purposes of analysis, the five-factor structure of PANSS developed by Wallwork, Fortgang [40] formed the basis of composite symptom scores: positive (items p1, p3, p5, a9), negative (items n1, n2, n3, n4, n6), disorganized (p2, n5, a11), depressive (a2, a3, a6) and excitatory (p4, p7, a8, a14). SCID1 interviews were also performed as part of the standard ward assessment routine, with diagnoses recorded according to ICD-10 criteria as per hospital policy. The PANSS assessments were performed by trained psychiatrists, with excellent inter-rater reliability (0.92).

### Statistics

Continuous variables were inspected using histograms to assess whether they conformed to the normal distribution. The percentage of participants showing cognitive impairment at baseline and follow-up was calculated, with the cut-off point for impairment being a total RBANS score of t < 35, i.e. > 1.5 SD below the mean score, corresponding to the xx impairment as conceptualised by Heaton and colleagues [41]. Univariate comparisons of cognitive scores at baseline and follow-up were done using two-tailed, paired t-tests, and Pearson correlation coefficients were used to analyse the relation between cognitive scores and PANSS scores at baseline. For all univariate analyses we applied Bonferroni-corrected alpha limits according to the number of comparisons or correlations involved, in order to avoid an inflated risk of Type 1 errors. Differences in the cognitive trajectory for subjects with schizophrenia spectrum disorders were assessed using repeated measures analysis of variance (ANOVA), with Greenhouse-Geisser corrections for violations of the assumption of sphericity. The grouping variable was constructed by dividing the patients into a Schizophrenia spectrum disorders group (SD; subjects given a primary diagnosis of F20, F23.0 or F23.2, i.e. fulfilling the core symptomatic criteria for F20, but not necessarily the duration criteria) vs. a group with other psychotic disorders.

Sequential multiple linear regression analyses with two blocks were conducted with RBANS change scores as the dependent variable to assess the contribution of symptom load change as measured by PANSS composite scores. In block one, the confounder variables gender, age, and baseline RBANS total and PANSS composite variable scores (Positive, Negative, Depressive, Excitatory and Disorganized) were entered, and in the second block, PANSS composite variable change scores were entered using forward stepwise selection (Criterion for inclusion: p < .05; criterion for exclusion: p < .1). Equivalent analyses were performed for change in each of the RBANS sub scales as well as for the RBANS composite score, with a p-value < 0.05 considered significant. Data were checked for multicollinearity, heteroscedasticity and normality of residuals. All analyses were conducted using SPSS 22 [42].

## Results

Table 2 shows the clinical and neurocognitive sample characteristics, as well as change in these domains from baseline to follow-up. The mean time from baseline to follow-up was 4.1 weeks (SD = 1.9 weeks). Diagnoses were drug-related psychosis (F12-19.9, n = 15, 17.9%), schizophrenia (F20-20.9, n = 20, 23.8%), schizotypal disorder (F21, n = 1, 1.2%), delusional disorder (F22-22.9, n = 10, 11.9%), acute psychotic disorders with symptoms of schizophrenia (F23.1-F23.2, n = 5), other acute psychotic disorders (without symptoms of schizophrenia) (F23.0, F23.3-F23.9, n = 17), other non-organic psychosis (F28, n = 1, 1.2%), unspecified non-organic psychosis NOS (F29, n = 6, 7.1%), and affective disorders with psychotic symptoms (F31-33.9, n = 9, 10.8%). In three patients, SCID was not performed, and the diagnosis from their discharge journal entry was used for the purposes of this study. There were 25 subjects (29.8%) with schizophrenia spectrum disorders and 59 subjects with other psychotic disorders (70.2%).

**Table 2:** RBANS and PANSS composite scores (n = 84).

	V1		V2		Δ V1 to V2			
	Mean	SD	Mean	SD	Mean	SD	p	E.S.^6^
**PANSS Measure** *Bonferroni-corrected alpha limit =* .*006*								
Positive	19.82	4.32	12.43	4.00	-7.39	4.75	<.001	1.78
Negative	18.37	7.07	15.24	6.50	-3.13	6.93	<.001	0.46
General	34.24	6.21	25.68	5.89	-8.56	7.15	<.001	1.41
Total score	72.43	12.72	53.35	13.58	-19.08	14.72	<.001	1.45
Comp. pos.^1^	3.32	.88	2.01	.85	-1.31	-1.31	<.001	1.51
Comp. neg. ^2^	2.48	1.19	2.18	1.13	-.30	1.21	= .025	0.26
Comp. dep. ^3^	3.14	1.04	2.26	.95	-.88	1.13	<.001	0.88
Comp. excit. ^4^	1.56	.58	1.20	.40	-.36	.63	<.001	0.73
Comp. dis. ^5^	2.52	1.08	1.82	.69	-.70	.88	<.001	0.79
**RBANS**^6^ **Score** *Bonferroni-corrected alpha limit =* .*008*								
Verbal	39.90	8.15	45.95	8.03	6.05	8.34	< .001	0.75
Visuo-spatial	46.25	12.65	47.86	10.97	1.61	10.20	= .152	0.13
Learning	36.58	10.77	38.54	10.72	1.95	9.69	= .068	0.18
Memory	40.29	11.27	40.10	12.38	-.19	11.08	= .875	0.02
Attention	30.32	8.80	33.93	9.57	3.61	7.44	< .001	0.39
RBANS mean	38.67	7.75	41.27	7.30	2.60	5.81	< .001	0.35

^1^. Composite positive symptoms score

^2^. Composite negative score

^3^. Composite depressive score

^4^. Composite excitatory score

^5^. Composite disorganized score

^6^. Repeatable Battery for the Assessment of Neurocognitive Status 6. Effect size (Cohen’s d)

### Neurocognitive and symptomatic change

**Fig 1:**
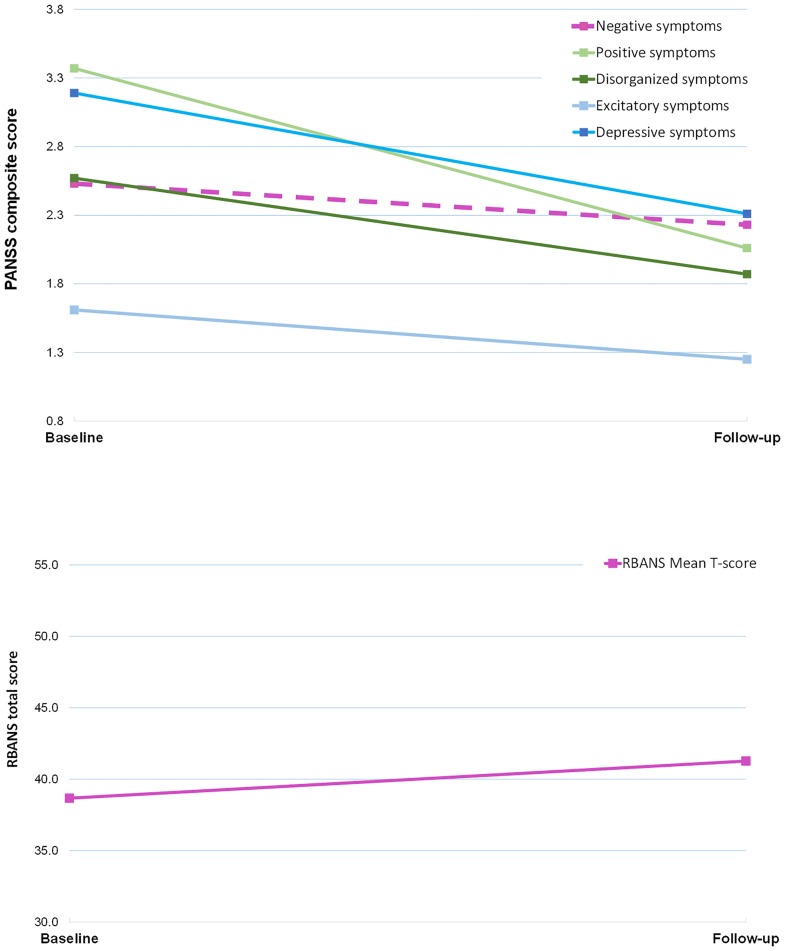
RBANS total and PANSS composite scores change baseline to follow-up.

Fig 1 shows mean RBANS total and PANSS symptoms at baseline and follow-up. There was a significant correlation (Bonferroni-corrected alpha level = .002) between baseline mean PANSS disorganized scores and RBANS total mean (r = -.515, p < .001), with a trend toward a relationship with Attention (r = -.296, p = .006) scores. No other symptom dimensions were significantly correlated with any RBANS scores.

At baseline, 28.6% (24/84) of patients were cognitively impaired with a total RBANS t-score of < 35, compared to 13.1% (11/84) of patients at the point of follow-up. There was a significant improvement in mean total RBANS scores from the mean of 38.67 at baseline to 41.27 at follow-up (p < .001, d = .35). Similarly, mean attention and verbal scores increased from 39.9 to 45.95 (p < .001, d = .75) and 30.32 to 33.93 (p < .001, d = -.39) respectively. A single-sample t-test showed that patients performed significantly worse than the normative average T-score of 50 both overall and for every RBANS subscale (all p-values < .001).

### Regression model of PANSS and RBANS change

The regression analysis, using RBANS composite change scores as the dependent variable, found change in negative symptoms to be the only significant predictor of improved RBANS performance (beta = -.307, p < .016). Details of the model are displayed in Table 3. Equivalent analyses were also performed for RBANS subscale change, but PANSS change scores were not a significant predictor of any of these scores.

**Table 3:** Regression analysis of PANSS change scores predicting RBANS change total (N = 84).

PANSS composite score	β	p
Positive	.124	.233
Negative	-.307	.016*
Depressive	-.121	.279
Excitatory	-.077	.464
Disorganized	-.060	.654

R^2^ = .305 p = .001. Adjusted for gender, age, and baseline PANSS composite variable scores

* Significant at p<.05

### Comparing schizophrenia spectrum disorders to other psychotic disorders

**Fig 2:**
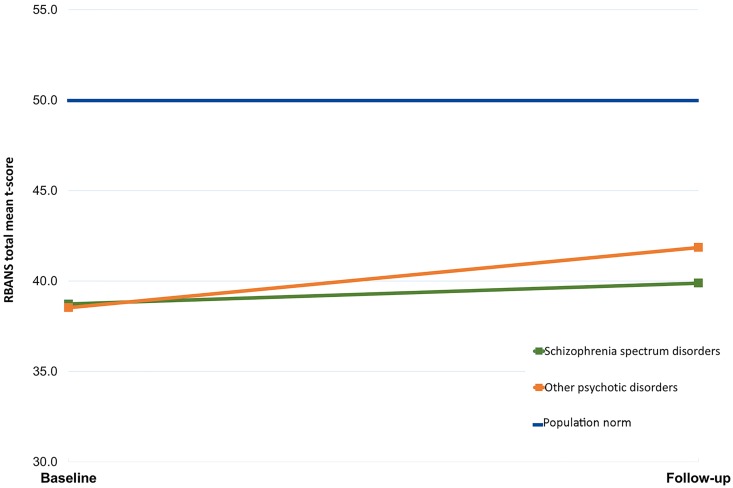
RBANS change from baseline to follow-up for schizophrenia spectrum disorders vs. other psychotic disorders.

A repeated measures ANOVA investigating the effect of age, gender and diagnostic group on cognitive change found no significant interaction between having a diagnosis of schizophrenia spectrum disorders, and time. Cognitive change for each group is displayed in Fig 2.

## Discussion

In line with our first hypothesis, we found that during the acute phase, 28.6% of patients performed below the normative sample, showing cognitive impairment defined as a T-score < 35, and the average T-score was 38.6, significantly lower than the normative mean of 50. Supporting our second hypothesis, total RBANS score increased significantly and the proportion of cognitively impaired subjects was reduced from 28.6% to 13.1% at the point of follow-up, four weeks later. Further, neurocognitive improvement was related to negative symptom improvement. Our third hypothesis was not supported, as subjects diagnosed with Schizophrenia spectrum disorders did not significantly differ from subjects with other psychotic disorders in terms of cognitive change.

The current findings indicate that significant improvement in cognitive functioning can take place quite early in the course of acute psychotic episodes. However, although positive symptom load decreased during the follow-up period, the improvement in neuropsychological test performance was in fact related to improvement of negative symptoms, despite these being generally less responsive to antipsychotic treatment than are positive symptoms [43]. The lack of a correlation with positive symptoms may seem counterintuitive, as a high positive symptom load might in itself be expected to be distracting, and thus associated with poor performance. Clinicians and researchers alike commonly postpone neuropsychological testing on this assumption, waiting for the clinical picture to stabilise [44].

The link between cognitive performance improvement and negative symptom improvement could be explained by avolition, i.e. decreased drive, which is a core characteristic of negative symptoms [43]. Drive, or the will to initiate goal directed behaviour, is in turn associated with motivation and effort to complete tasks. Impaired effort and amotivation have indeed been found related to negative symptoms, and to account for about a third of variance in cognitive test performance in schizophrenia [31, 45]. Subjects with schizophrenia have been found to show attenuated brain responses to rewards, which may be partly driving impairments in motivation [46]. A proportion of the improved performance seen in our sample could thus be accounted for by improved motivation and effort as a result of receding negative symptoms, in line with previous findings showing that 9.1% of subjects with schizophrenia fall below the effort indicator cut-off for RBANS [47].

Neurocognitive impairment in schizophrenia has commonly been understood as a purported trait rather than a state phenomenon [48, 49], with positive symptoms assumed to be related to temporary state impairment. State effects of psychosis, positively correlated with positive symptom load, have been seen for instance in the case of theory of mind [50], and depth perception [51]. The fact that cognitive change in our study was unrelated to both baseline positive symptoms and the alleviation of these indicates however that the change does not simply reflect amelioration of a state effect caused e.g. by intense hallucinations at baseline testing. This means that clinicians should perhaps worry less about positive symptoms being a threat to the validity of neurocognitive assessment, but be more aware of negative symptoms which might affect test performance.

This is to our knowledge the first study to show a correlation between amelioration of negative symptoms during acute psychosis and improved cognitive functioning in a group including patients with psychotic relapse. With cognitive impairment being a central element of psychotic illness, it is possible that cognitive symptoms fluctuate alongside positive, negative and disorganized symptoms, with negative symptoms being the most closely related symptom dimension. Our findings indicate that positive changes in cognitive function are seen in heterogeneous groups of subjects with psychosis, not simply in homogenous FEP samples.

Our findings, with an omnibus total R squared of .305, indicate in line with previous research that negative symptoms account for only a limited proportion of variance in change of cognitive impairment [52]. However, we have found that cognitive impairment does appear to change in parallel over time with negative symptoms. PANSS negative symptoms and RBANS total scores were significantly correlated at baseline, but the absolute change in negative symptoms, rather than baseline severity, appears to be the key factor for improved cognitive performance. Although this does not on its own indicate a causal relationship in either direction, it may be seen as an indication that cognition and negative symptoms are closely associated with each other.

Although they are separate constructs, cognitive impairment and negative symptoms appear linked in that they both occur before the development of positive symptoms, have shared courses and prognostic importance [53]. Neither responds to antipsychotic treatment to the same extent as do positive symptoms [43]. Negative and cognitive symptoms might also be related to structural and functional abnormalities in the same prefrontal areas. Cognitive impairment in schizophrenia has been tied to hypofrontality during task performance, in the form of bilaterally reduced activation in the dorsolateral prefrontal cortex, compared with both cognitively non-impaired individuals and healthy controls [54]. Negative symptoms have been associated with reductions in dorsolateral prefrontal grey matter [55], as well as prefrontal white matter [56]. Our findings could be seen as supporting a two-pathway model of psychosis in which a negative and disorganized dimension is associated with a phenotype of neurocognitive impairment, which in turn is unrelated to positive and affective symptom dimensions [19].

Interestingly, although PANSS disorganized scores were also significantly correlated with RBANS total mean, disorganized symptom change failed to significantly explain changes in performance at follow-up. This was despite average disorganized symptom change having an effect size of .079, compared to .026 for negative symptoms. The correlation between total RBANS scores and disorganized score could possibly be explained by the inclusion of PANSS items n5 (problems with abstract thinking) and a11 (poor attention) in the disorganized composite score. Both of these may to some extent also be measured by neurocognitive assessment.

There were no differences in cognitive change between patients with Schizophrenia spectrum disorders and those with other psychotic disorders. This is contrary to previous findings [10] which have looked at change in groups with schizophrenia alone, i.e. having multiple symptoms of psychosis across dimensions, which over a longer period of time have caused functional distress.

### Strengths

With baseline neurocognitive testing for the Bergen Psychosis project performed immediately after hospital admission (in most cases within 48 hrs), this study is one of few investigating cognitive change in the very early acute phase of illness, gathering baseline data while subjects were actively psychotic and re-testing them quite shortly after remission began. Our findings thus provide important information about the early time course of cognitive abilities in psychotic episodes, especially given the paucity in this field of data gathered during the early acute phase. The short test-retest interval in the inpatient setting also means this study was less vulnerable to attrition than are studies with a longer retest interval. Finally, the patients were included consecutively, increasing the representativeness of the sample.

### Limitations

No control group was included in this study, precluding statistical control of the changes seen in patients with learning effects in healthy individuals. Familiarity with procedures and practice might have affected the results. However, the RBANS neurocognitive battery is designed to withstand such effects. The exclusion of patients unable to cooperate during testing might have made results less representative of subjects with acute psychosis. Most exclusions happened on the basis of patients acting out or being unable to cooperate during assessment, or them being ineligible for oral antipsychotic medication.

## Conclusions

In our study, the percentage of cognitively impaired subjects fell from 28.6% during acute phase treatment to 13.1%, at follow-up, demonstrating the potential for positive change in this symptom dimension. Our findings that cognitive improvement is related to improvement in negative symptoms highlight the importance of monitoring the trajectory of negative symptoms across the acute phase of illness. Often less obvious to the clinician than residual positive symptoms, lack of change in negative symptoms might be an important indication of persistent cognitive impairment. Even after the initial deterioration, cognitive recovery is possible and does happen with appropriate treatment and negative symptom remission.
